# Implicit spatial sequential learning facilitates attentional selection in covert visual search. An event-related potentials study

**DOI:** 10.3389/fnhum.2022.974791

**Published:** 2022-12-01

**Authors:** Marta Szewczyk, Paweł Augustynowicz, Magdalena Szubielska

**Affiliations:** Perception and Cognition Lab, Department of Experimental Psychology, Institute of Psychology, The John Paul II Catholic University of Lublin, Lublin, Poland

**Keywords:** selective attention, visual search, implicit learning, spatial predictability, temporal predictability, sequential learning, N2pc

## Abstract

**Introduction:**

While most studies on implicit sequential learning focus on object learning, the hidden structure of target location and onset time can also be a subject of implicitly gathered knowledge. In our study, we wanted to investigate the effect of implicitly learned spatial and temporal sequential predictability on performance in a localization task in a paradigm in which covert selective attention is engaged. We were also interested in the neural mechanism of the facilitating effect of the predictable spatio-temporal context on visual search processes. Specifically, with the use of an event-related potential technique, we wanted to verify whether perceptual, attentional, and motor processes can be enhanced by the predictive spatio-temporal context of visual stimuli.

**Methods:**

We analyzed data from 15 young, healthy adults who took part in an experimental electroencephalographic (EEG) study and performed a visual search localization task. Predictable sequences of four target locations and/or target onset times were presented in separate blocks of trials that formed the Space, Space- Time, and Time conditions. One block of trials with randomly presented stimuli served as a control condition.

**Results:**

The behavioral results revealed that participants successfully learned only the spatial dimension of target predictability. Although spatial predictability was a response-relevant dimension, we found that attentional selection–instead of motor preparation–was the facilitation mechanism in this type of visual search task. This was manifested by a shorter latency and more negative amplitude of the N2pc component and the lack of an effect on the sLRP component. We observed no effect of predictability on perceptual processing (P1 component).

**Discussion:**

We discuss these results with reference to the current knowledge on sequential learning. Our findings also contribute to the current debate on the predictive coding theory.

## Introduction

Many events in our everyday lives occur sequentially. During a single year, daytime varies in length for each day, but the pattern–a sequence of 365 elements–repeats every year. A typical car driver is used to the fact that green traffic light appears shortly after the red and the amber are lit together. Then, the green light stays on for slightly longer, until the amber light appears briefly, followed by a longer red. These are commonly known examples of temporal sequences. The Sun’s position in the sky, as well as the traffic lights’ placement, are also spatially sequenced. For instance, we always expect to see the red light in the top part of the traffic signal, the amber in the middle, and the green light at the bottom. According to the predictive coding theory, our brain is equipped with a mechanism for detecting repetitive patterns in order to facilitate action in predictable circumstances. The need to better understand the role of spatiotemporal sequential predictability in human cognitive functioning became the inspiration for this study.

### Sequential learning paradigm

Sequential learning (SL) refers to the learning of structured patterns of stimuli presented in a non-random order (Conway and Christiansen, 2005; Conway, 2012). The most popular paradigm for studying SL in the spatial domain is the serial reaction time task (SRTT), which was proposed by Nissen and Bullemer (1987). This task usually consists of localizing single targets that appear at four different locations. The sequence of locations is predictable, but this fact is not revealed to the participants. The more time that participants spend on this task, the more their reaction times decrease. One of the last blocks contains an interrupted sequence to differentiate the SL effects from the task learning effects (i.e., decreasing reaction time with increasing time on task). If the reaction time for this new (unpredictable) sequence increases, this shows that the participants have successfully learned the repetitive pattern and are surprised by the new sequence. The paradigm has been further developed to test not only on a specific stimuli features’ predictability, but also the predictability of a task set (see e.g., Heuer et al., 2001; Kleinsorge et al., 2003).

In a recent review, Conway (2020) proposed a unified theory of statistical learning and outlined its ten principles. In this view, sequential learning is considered one of three paradigms for studying statistical learning. The other two are artificial grammar learning (Reber, 1967) and the word segmentation task (Saffran et al., 1996; Fiser and Aslin, 2001). In contrast to previous conceptualizations (cf., e.g., Daltrozzo and Conway, 2014), the unified theory (Conway, 2020) incorporates orthogonal dimensions that underlie the construct of statistical learning. These dimensions are: (1) the level of structure present in the sequences; (2) the amount of exposure to the sequenced information (3) the amount of explicit instructions or overt feedback provided by the task situations. Ideal statistical learning occurs when the following conditions are (jointly) met: the information/input is highly structured, the exposition is sufficiently long or intensive, and there is no overt instruction or feedback provided. Though the author stipulates that the coincidence of all these criteria is rarely possible, mapping a specific situation onto these three dimensions may help to determine what other factors should be considered for a successful acquisition of implicit knowledge (e.g., how much working memory or attentional resources are needed for a cross-modal SL). The model comprises two learning systems: implicit (attention-independent) and explicit (attention-dependent). They potentially act competitively, although, under certain conditions, they may also cooperate. Detailed reference to all ten principles is beyond the scope of this article, although some of them provide essential background for our research problem.

According to the unified theory (Conway, 2020), cortical plasticity and top-down modulatory control are the two mechanisms that govern statistical learning. In this regard, cortical plasticity enables perceptual and associative learning. It is “an ever-present and obligatory mechanism, instantiated over multiple, hierarchically-embedded networks” (Conway, 2020, p. 293). The top-down executive system is responsible for learning more complex patterns. It recruits attentional and working memory resources (Fuster and Bressler, 2012; Hasson et al., 2015), localized in the prefrontal cortex. Conway (2020) postulates that the two mechanisms operate independently and in parallel. Prediction and expectation–regarded as fundamental human cognitive functions (Friston, 2005; Dale et al., 2012), are the key triggers of statistical learning. Additionally, “learning can occur for a variety of input structures that vary in complexity” (Conway, 2020, p. 293). Within this perspective, space and time can be equally regarded as important dimensions of predictability as other types of input.

### Nature of sequential learning

Numerous pieces of research on implicit sequential learning that were conducted in the SRTT paradigm have sought answers concerning the nature of SL: is it perceptual, motor or hybrid? (Praamstra et al., 2006; Abrahamse et al., 2010; Schwarb and Schumacher, 2012; Guo et al., 2013; Verwey et al., 2014). However, only a few studies have addressed the nature of sequential learning in the spatial and temporal domains. Howard et al. (1992) suggested that spatial SL is primarily based on perceptual learning. In turn, Mayr (1996) argued that SL depends on both perceptual and motor processes. The author concluded that perceptual processes–especially attentional control of visual stimuli–and motor processes, i.e., response selection, are engaged in SL equally but independently. In other words, each type of sequential learning can be triggered alone (i.e., regardless of the presence of another dimension of predictability), but each type of SL independently contributes to the overall learning benefits. In one of their two experiments on motor sequence learning, Willingham et al. (2000) found evidence of motor learning but with regard to the cognitive process of response selection and not the muscular process of movement execution. Based on a literature review, Schwarb and Schumacher (2012) stated that the mechanism of spatial sequence learning is stimulus-response (S-R) associative learning. In order to effectively gain knowledge on a specific sequence, the process of response selection is crucial. Nevertheless, not all studies allow such a conclusion. Associative learning of S-R mapping is not engaged in the learning process in the auditory domain, especially when selective attention is at play (see: Goschke, 1998). According to Abrahamse et al. (2010), sequential learning is not limited to one specific type of information but gradually emerges as a result of processes activated by specific task demands. The representation that participants acquire in the process of incidental learning is the regularity of the events that are part of the ongoing task. Thus, building associations between subsequent stimuli’s features is just as possible as both building associations between reactions’ features and linking stimuli’s and reactions’ features.

### Spatial and temporal dimensions of predictability

Another important yet unanswered question is whether the spatial and temporal dimensions of predictability act independently or in synchrony. This issue was examined by O’Reilly et al. (2008), who used the localization task in the no-search SRTT paradigm. Although temporal predictability did not have an independent effect on the efficiency of the localization task, it significantly improved the facilitation effect of spatial predictability. O’Reilly et al. (2008) conclude that implicit learning of temporal sequences requires the presence of motor predictability, i.e., reaction predictability. Nevertheless, they point to instances when learning of complex rhythms occurs with reference to sequences not necessarily correlated with a specific action. For instance, the rhythm of a musical composition can be reproduced with a hand, a foot, or any other part of the body. Shin and Ivry’s (2002) study aimed to verify whether (1) a temporal sequence can be implicitly learned together with a motor sequence, i.e., with a spatial sequence in a localization task; (2) whether a temporal sequence can be implicitly learned independently from sequential motor learning; and (3) whether the presence of a temporal sequence facilitates sequential motor learning. The results of their experiment showed that knowledge of the spatial sequence was acquired regardless of whether the sequence was presented in isolation or in combination with the temporal sequence. The temporal sequence was learned only when it was presented together with the spatial sequence. The authors conclude that acquisition of implicit knowledge about the hidden temporal structure of the presentation of visual stimuli primarily serves motor facilitation but not perceptual facilitation, since the effects of temporal sequential predictability were only observed when knowledge about a predictable structure was useful for target localization, but not when the elements of a given sequence were unrelated to any specific reaction. Shin and Ivry (2002) further speculate that a sequence of eight elements was too long to be integrated as a unified mental representation of a predictable whole. Moreover, conclusions based on simple chronometric analysis with no neuroimaging data are limited to motor performance, while little can be said regarding the early perceptual and attentional processes. A similar study by Heideman et al. (2018) showed that target localization was significantly faster for spatially and temporarily predictable stimuli than with randomly presented targets. What is more, a specific pattern of the behavioral costs of a sequence change was observed mainly for short intervals. In other words, the reaction time difference (between temporally predictable and unpredictable targets) was bigger for short intervals since the predictability of the long intervals was always high on the basis of the hazard function (i.e., the probability of a target appearing increases with increasing waiting time).

Studies (Shin and Ivry, 2002; O’Reilly et al., 2008; Heideman et al., 2018) consistently show that temporal predictability can be implicitly learned only when high spatial predictability is present. With the use of magnetoencephalography (MEG), Heideman et al. (2018) also showed that beta suppression in the motor cortex areas is the neural mechanism that drives motor facilitation in predictable circumstances. These authors emphasize the similarity of this result to the outcomes of studies where predictability is elicited by external symbolic cues. However, since the authors did not test a condition of single spatial or temporal predictability, their experiment does not make it possible to state whether the effects of sequential predictability in temporal and spatial domains are interdependent or whether they can form a synergy effect. Such a synergy would consist of a stronger facilitation (bigger reaction time benefits) of spatio-temporal predictability than unidimensional predictability. This synergy effect was nevertheless detected by O’Reilly et al. (2008) and Shin and Ivry (2002). Heideman et al. (2018) note that in comparison to the effect of spatio-temporal predictability on motor preparation and response execution, a relatively small effect is observed with relation to perceptual and attentional processes. This, in turn, is typical of the predictability evoked by external symbolic cues or by a constant rhythm. Thus, given the limitations of the previous studies, there is a need to further explore the interplay between the spatial and temporal dimensions of predictability and their influence on perceptual, attentional, and motor processing.

### Spatial predictability in the visual search paradigm

Three studies on spatial predictability in the visual search paradigm were done by McDonnell et al. (2014) and Coomans et al. (2011, 2014); note that predictability in two of these studies, [i.e., McDonnell et al. (2014) and Coomans et al. (2011), was not based on sequential learning]. The results obtained by Coomans et al. (2011) are important for our research question as they provide some insight into the functioning of implicit learning in visual search tasks. The authors focused on the effect of perceptual load on the efficiency of acquiring and expressing implicit knowledge about a predictable target’s location. Spatial predictability was based on probabilistic sequential learning, meaning that a target in trial *n* could appear in the same place as in trial *n*-1 or in a different but non-random place. Participants had to discriminate between two types of targets: XO vs. OX. Three other locations contained distractors representing a low (MN, NM) or high (QX, XQ, YQ, QY) perceptual load. The first experiment showed that no transfer effect was observed in conditions of no perceptual load in the testing blocks. Although reaction time gradually decreased as time on the task increased, reactions in the block, which contained a new sequence, were not significantly longer. The second experiment revealed that the effects of implicit learning are only seen in conditions of high perceptual load in the testing phase. The third experiment showed that acquisition of implicit knowledge does not require any perceptual load.

McDonnell et al. (2014) wanted to determine whether the pattern of oculomotor reactions plays a crucial role in the learning of a predictable spatial structure of visual stimuli presentation, or whether this type of implicit learning affects visual search processes independently from eye movements. The facilitation mechanism might, for instance, affect the response criterion. The authors used a detection task in which the position of the target, which was presented amongst distractors, was predictable based on the previous target position. Unsurprisingly, participants reacted faster in the spatially predictable context. However, faster reactions did not result from faster first saccades directed toward the part of the visual field where the target was most expected. Similar results were observed by Beck et al. (2014) with regard to the predictable frequency rule. Coomans et al. (2014), based on their study on implicit learning in the visual search paradigm, found that eye movement is not necessary for implicit knowledge consolidation. Since all these studies contradict the oculomotor facilitation hypothesis, they may support the perceptual and attentional speed-up account. More direct investigation of the facilitation mechanism of predictable stimuli processing is possible with the use of the event-related potential (ERP) technique. The specific electroencephalographic indexes of each stage of stimulus processing are outlined in the next paragraphs.

### Event-related potentials related to different stages of stimulus processing

The P1 component is a positive deflection recorded from the occipital cortex. It has an onset latency of approx. 60–100 ms, and is considered an electrophysiological correlate of the early sensory stage of visual stimuli analysis (Mangun and Hillyard, 1991). It shows sensitivity to the involvement of spatial attention–its amplitude is respectively higher or lower in response to stimuli presented in an attended or unattended part of the visual field. Luck et al. (1990) showed that the P1 is associated with facilitation of the early stages of sensory analysis of a stimulus, starting even before its occurrence. Thus, P1 reflects the directing of attention to a given part of the visual field in anticipation of the upcoming stimulus (see also Heinze et al., 1990). If perceptual processing is in fact facilitated by implicit spatial and temporal SL, we expect to observe shorter latency and higher amplitude of the P1 component for stimuli following the predictable spatial, temporal, and spatio-temporal sequence(s).

The N2pc component is an electrophysiological correlate of the attentional selection process, acquired as a difference wave between the activity recorded from the contralateral and ipsilateral electrodes, respective to the target-containing part of the visual field. Such a difference wave makes it possible to obtain a component that is free from the contamination of other non-lateralized components, such as P3 or CNV (Luck, 2012). It is recorded from the parieto-occipital cortex (PO3/PO4, PO7/PO8, O1/O2 electrodes) in the time window that is approx. 200–300 ms after the presentation of a set of visual stimuli. Its onset latency is shorter for more salient stimuli (Luck et al., 2006) and correlates positively with reaction times, although it does not fully determine them (Wolber and Wascher, 2005). Unlike bottom-up visual components (e.g., P1), even highly salient stimuli do not trigger the N2pc component if they are task-irrelevant (Luck and Hillyard, 1994). This proves that N2pc is a correlate of top-down spatial selective attention; it is not just a side effect of the asymmetrical processing of physically different stimuli (Eimer, 2013). Töllner et al. (2012a) showed that the prevalence of a specific target affects the attentional selection process in the detection task in a visual search paradigm. The amplitude of the N2pc component was less negative for frequent as compared to infrequent targets. In a study that manipulated the predictability of target modality (visual vs. acoustic), a more negative N2pc amplitude was observed for visual predictable stimuli compared to the amplitude for unpredictable visual stimuli (Töllner et al., 2012b). Additionally, longer N2pc latency was observed for unpredictable compared to predictable targets with a short (150 ms) time interval and shorter latency of N2pc for predictable compared to unpredictable targets with a long (600 ms) time interval. However, neither of these two studies manipulated the predictability of the target’s location or onset time. It remains an open question whether the attentional selection process is facilitated by predictability in the spatial and temporal dimensions in a similar way that it is facilitated by the predictability of other dimensions of stimuli’s features.

Another potential mechanism of the influence of spatio-temporal predictability on the localization of visual stimuli may be related to motor processes, i.e., response selection, as measured by the lateralized readiness potential (LRP). This component is recorded from the sensorimotor cortex area (C3/C4 electrodes), ipsilateral to the reaction-executing hand. Similarly to N2pc, it is subtracted from the more negative potential recorded by the electrode contralateral to the responding hand (Coles, 1989). In this view, the LRP component is an indicator of the extent to which one hand is more activated than the other during the reaction execution. The time between the presentation of the stimulus and the onset of the LRP component is considered the stimulus processing time, as lateralization of the electrophysiological response in the form of the LRP wave is not possible until the motor cortex receives a signal concerning which hand should react in a given trial. Thus, the LRP onset latency reflects the time needed to register a stimulus, recognize it, and initiate the reaction-selection process (Hackley and Valle-Inclán, 2003). Consequently, the LRP component calculated with respect to the timing of stimulus exposure is termed sLRP (stimulus-locked LRP) and is interpreted as a response selection correlate. Even though it is recorded from the same electrodes as the N2 and P3 components, LRP is free from their contamination thanks to the use of the subtraction technique (Luck, 2012). The LRP amplitude depends on the complexity level of the prepared reaction (e.g., Hackley and Miller, 1995), but not on its strength (e.g., Sommer et al., 1994) or the task in which it is to be performed (Miller and Low, 2001). It has been shown that the sLRP component is sensitive to target intensity (Miller et al., 1999) and the quality (Smulders et al., 1995) of visual stimuli. Visual objects that are attentionally prioritized because of their selection history are also associated with faster response selection. In the study by Töllner et al. (2012a), the high predictability of a specific target type evoked an sLRP with shorter latency and smaller amplitude (when compared to the less probable targets). This may indicate that a lowered decision threshold is needed for a specific reaction to be performed (Chun and Wolfe, 1996). In other words, high predictability means that the cognitive system needs little perceptual evidence in order to make a decision about the reaction required in a given trial. More evidence would be needed to react to targets whose key features are less predictably distributed. Therefore, if the lowered response selection threshold phenomenon concerns not only the predictable target type (Töllner et al., 2012a) but also the spatio-temporal structure of its exposure, targets presented in accordance with the subject’s expectations should trigger sLRP with a shorter latency and lower amplitude than randomly presented stimuli.

### The present study

Although sequential learning has been extensively studied, it does not cease to inspire new research questions. Previous research has consistently shown that implicit sequential learning does not occur for a single temporal dimension in a no-search paradigm. However, the problem of predictable sequential learning in temporal and spatio-temporal dimensions in a visual search paradigm has not yet been addressed. High perceptual load can create optimal conditions for implicit knowledge to be expressed, since there are indications that this type of knowledge might only be relevant in such demanding conditions (Navon, 1979; Coomans et al., 2011). Therefore, it is crucial to know more about the (in)dependence of a predictable sequence in spatial and temporal dimensions in a visual search task.

Previous studies on the mechanism of sequential learning found that it mainly involves S-R associations (Schwarb and Schumacher, 2012), although other mechanisms can be also engaged (c.f., Abrahamse et al., 2010), especially when selective attention is at play (e.g., Goschke, 1998). The learning of spatial sequences is driven by perceptual learning and occurs even without predictability in all other dimensions (e.g., temporal or related to a target feature, see Mayr, 1996). On the other hand, the learning of temporal sequences depends on motor processes since knowledge about a sequence is observed only when a repetitive stimulus-reaction association is established (e.g., Shin and Ivry, 2002; O’Reilly et al., 2008). In such cases, temporal predictability enhances the effect of spatial predictability (Shin and Ivry, 2002; O’Reilly et al., 2008; Heideman et al., 2018).

What we wanted to explore in our research is not the mere mechanism of implicit spatial and temporal learning but the facilitation mechanism of predictable stimuli processing when knowledge of the predictable spatio-temporal structure of stimuli occurrence has already been acquired. To our knowledge, no previous studies have yet comprehensively tackled this issue. A study by Goschke (1998) suggests that the mechanism of sequential learning with distractors is different from sequential learning in the single stimulus exposition paradigm. Since Goschke’s findings concerned the auditory modality, the functioning of visual processes in a predictive spatio-temporal context is still an open question. Therefore, the aim of the present study was to verify whether a predictable order of a target’s place and time of appearance facilitates its localization in a visual search task. Additionally, by using the ERP technique, we wanted to determine the stage of the facilitated processing, i.e., whether this type of implicit knowledge about the structure of a target’s exposure enables faster perception, attentional selection, or response-related processes.

As regards the hypotheses, on the behavioral level we expected faster reactions for spatially, temporally, and spatio-temporally predictable stimuli, compared to the randomly exposed stimuli. Additionally, based on the O’Reilly et al. (2008) and Shin and Ivry (2002) findings, we expected to observe a larger facilitation effect caused by the spatio-temporally targets, in comparison to the stimuli predictable in one dimension. To this end we used a paradigm in which participants were supposed to implicitly learn short (four-element) sequences of a target’s location and/or target onset time while performing a visual search localization task. We tested each dimension of predictability in a separate block of trials. In order to separate the processes that underlie the stage of acquiring implicit knowledge from the processing stage, when this knowledge could already serve as an attentional cue, we divided trials in each block into the learning phase and the testing phase (c.f., Schwarb and Schumacher, 2012). To verify the effectiveness of implicit learning, the testing phase was followed by trials with a broken sequence (c.f., Coomans et al., 2011). The old predictable (i.e., recovered) sequence was presented in the final trials of each block. Effective implicit learning should be manifested by a reaction time decrease throughout the learning and testing phases, followed by a reaction time increase for stimuli in the “broken sequence” phase, and then by a reaction time decrease when the old predictable sequence reappears (c.f., Coomans et al., 2011).

With reference to the facilitation mechanism, on the basis of studies showing that SL relies on perceptual learning (e.g., Howard et al., 1992; Mayr, 1996), we expected enhanced perceptual processing for spatially and temporally predictable stimuli. In terms of electrophysiological measures, enhanced perceptual processing should be exhibited as shorter latency and higher amplitude of the P1 component. Additional support for this hypothesis comes from research that showed no evidence for oculomotor learning in a predictable spatio-temporal context (Coomans et al., 2011, 2014; McDonnell et al., 2014). These studies also suggest that attentional selection might be facilitated by the presence of spatial or temporal predictability. As the N2pc component is a well-established ERP correlate of attentional selection, we expected to detect its shorter latency and enhanced amplitude for spatially and temporally predictable targets. Since motor learning is another important component of implicit sequential learning (especially in the temporal domain), we expected speeded motor processing (i.e., faster response selection manifested in the form of shorter latency of the sLRP component) for spatially and temporally predictable targets (cf., Mayr, 1996; Willingham et al., 2000; O’Reilly et al., 2008; Heideman et al., 2018). Since P1 is a visual component, spatial more than temporal predictability should facilitate this early perceptual stage of processing. Based on the findings by Töllner et al. (2012b) and Töllner et al. (2012a), we expect a similar facilitation of attentional selection (N2pc component) by both spatial as well as the temporal domain. Response selection (sLRP component), on the other hand, will hypothetically be more affected by temporal than spatial predictability (c.f., Shin and Ivry, 2002; O’Reilly et al., 2008; Heideman et al., 2018). Only stimuli from the testing phase were included in the electroencephalographic analyses.

## Materials and methods

### Participants

*A priori* power analyses using G*Power Software revealed that 10 participants would be needed to detect a within-participant effect in a repeated measure analysis of variance (rmANOVA) with one group and four comparisons (Random vs. Space vs. Space-Time vs. Time blocks), assuming large effect size (*f* = 0.40, which equals to the η_p_^2^ = 0.14), significance level of *p* < 0.05, and a power of 0.80. Taking into account that some participants could be excluded from the analyses because of the excessive electroencephalographic (EEG) artifacts, our sample initially included 20 participants (aged 20 to 28 years). They were recruited *via* an on-line questionnaire published in local social media groups. All participants were paid 50 PLN (ca. 11 euros) for their contribution. Six participants were excluded from the analyses due to noisy EEG data. The final sample included 14 subjects aged 20 to 26 years (M = 22.13; SD = 2.07). All participants gave an informed consent prior to participation. The study was conducted in accordance with the ethical standards of the Declaration of Helsinki (1964).

### Stimuli and task

**FIGURE 1:**
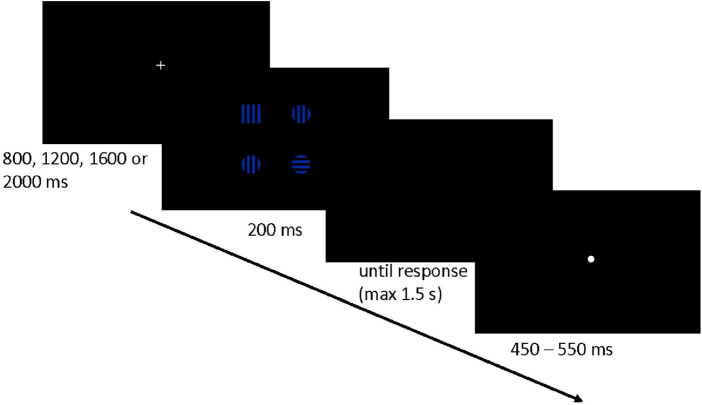
The schema of the trial flow (For ease of the presentation, the size of the stimuli in the picture does not correspond to their real size on the computer screen).

**FIGURE 2:**
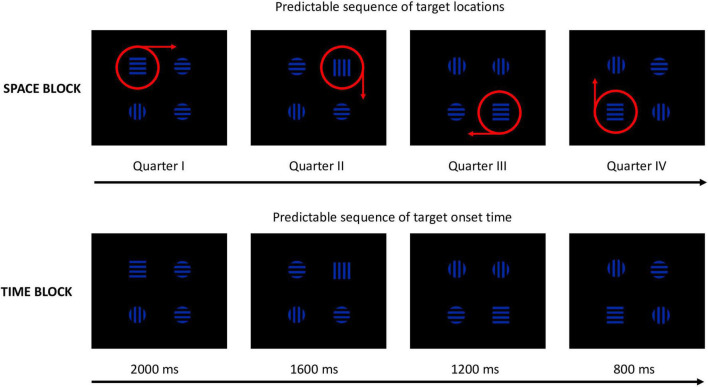
Examples of the spatial and temporal sequences (The red circles and arrows were not displayed on the screen).

The set of stimuli consisted of one target square and three distracting circles. All stimuli (sized 2.4*^o^* × 2.4*^o^* of the visual angle, see Figure 1) were filled with black and blue horizontal or vertical stripes [0.3*^o^* of the visual angle, Red Green Blue (RGB) values for blue (0 0 164), for black (0 0 0)]. The stimuli were displayed simultaneously 4.6*^o^* from the center of the screen against a black background in such a way that each shape was placed in one of the four corners of an imaginary square. The stripes’ orientation changed randomly across trials, independently for each stimulus (see Figure 2). Half of the stimuli contained vertical stripes, and the other half contained horizontal stripes.

Participants had to press the left/right key of the keyboard (A/L letter key) with their index fingers if they saw a target square located in the left/right part of the screen, respectively. Each trial began with a presentation of a white fixation cross (0.76*^o^* × 0.76*^o^*). After a variable time period–depending on the experimental condition–a set of four stimuli (i.e., one target square and three distracting circles) was displayed for 200 ms. Right after participants gave a correct response (or after 1,200 ms in the case of no reaction), a white dot (0.67*^o^*) appeared in the center of the screen, and the next trial began (see Figure 1 for an exemplary trial flow visualization). The stimuli presentation time (i.e., 200 ms) was intended to prevent saccadic eye movements. In the case of an erroneous response, a red minus sign (0.80*^o^*) appeared in the center of the screen for 1,500 ms.

### Design

The task consisted of four big blocks: Space, Space-Time, Time and Random. In the three big predictable blocks (Space, Space-Time, Time), the target’s place, onset time or place and onset time were predictable based on the rule explained below. The fourth big block was a control condition, in which the target’s place and onset time were pseudo-randomized. Each big predictable block was further sub-divided into ten mini-blocks (48 trials in each). The random block consisted of six mini-blocks (48 trials in each). In total, there were 1,728 trials in the whole experiment: 480 trials in each big predictable block and 288 trials in one random block.

Spatio-temporal sequential predictability was implemented with the use of sequences of target locations and onset times. Each sequence consisted of four elements: e.g., in the Space block, the first target appeared in quarter I, the second target appeared in quarter II, the third target appeared in quarter III, and the fourth quarter appeared in quarter IV. The fifth target appeared again in quarter I, and all subsequent stimuli followed the same order. To prevent learning of spatial and temporal sequences between blocks, the combinations of sequences were counterbalanced in such way that participants with odd numbers were presented with the following sequence of target locations in the Space block: quarter I–quarter II–quarter III–quarter IV, and the following sequence of target locations in the Space-Time block: quarter IV–quarter III–quarter II–quarter I. In the Time block they were presented with the following sequence of TOTs: 800–1,200 ms, 1,600–2,000 ms (see Figure 2), while in the Space-Time the TOTs followed the sequence: 2,000–1,600 ms, 1,200–800 ms. Participants with even numbers were presented with the following sequence of target locations in the Space block: quarter IV–quarter III–quarter II–quarter, and the following sequence of target locations in the Space-Time block: quarter I–quarter II–quarter III–quarter IV. The sequence of TOTs for these participants in the Time block was the following: 2,000–1,600 ms, 1,200–800 ms, and in the Space-Time block TOTs followed the sequence: 800–1,200 ms, 1,600–2,000 ms. These sequences were repeated in a loop. Twelve repetitions of a sequence formed one mini-block (48 trials). After each mini-block, there was a 15-second break. After three mini-blocks (i.e., after 144 trials), participants could take a longer break, the duration of which was dependent on their needs. The second round of three mini-blocks was followed by three mini-blocks of trials with a broken sequence. The first two elements of the broken sequence were identical to those from the standard sequence (e.g., quarter I–quarter II in the Space block), but the last two elements were different (e.g., quarter IV–quarter III) and were randomly changing from trial to trial. After each mini-block with a broken sequence, there was a 15-s. break. The last mini-block with a broken sequence was followed by one mini-block with the standard predictable sequence. The three mini-blocks with a broken sequence were supposed to evoke a reaction time increase when compared to the standard predictable blocks due to the surprise effect. The last mini-block with the old predictable sequence should in turn evoke a reaction time decrease relative to the reaction times in the broken mini-blocks (cf. Coomans et al., 2011). The above-described design (2 × 3 mini-blocks with predictable sequence, 1 × 3 mini-blocks with a broken sequence and one mini-block with the old predictable sequence) was only applied for the big predictable blocks, i.e., Space, Space-Time, and Time. The control block consisted of six mini-blocks (x 48 trials) in which the stimuli were exposed randomly but with equal probability of each target’s location and TOT occurrence. This procedure was designed to, first, enable implicit sequential learning during the first three mini-blocks (i.e., 36 repetitions of a four-element sequence); second, to compare the indexes of attentional functioning in mini-blocks 4–6 between predictable blocks vs. the control block; third, to distinguish between the effects of implicit learning and the task learning effect.

### Procedure

All participants were tested individually. They were comfortably seated in a dimly lit experimental room at a distance of ca. 65 cm from the computer screen. After the EEG cap montage and impedance adjusting, they read the instructions displayed on the computer screen and performed a short training session (30 trials of a random block) to get familiar with the localization task. 90% accuracy was needed to proceed to the main task. Participants were instructed to keep a stable body and head position during the task and to use between-block breaks to have a rest. They were additionally asked to rely on their peripheral visual field to perform the visual search task, i.e., to keep central gaze fixation and to avoid any eye movements which could distort the EEG data. The experiment took approximately 90 min, excluding time for the cap preparation, impedance adjustment and instructions (ca. 45 min).

### Apparatus

The electroencephalogram was recorded with the use of a 64-channel EEG system (ActiCap system, Brain Products, Munich, Germany) and an amplifier by Electrical Geodesic Inc. (Eugene OR, USA). The stimuli were displayed on a 24-inch computer screen (refresh rate 60 Hz, resolution 1,920 × 1,080). The experimental procedure was written in PsychoPy. NetStation 4.4 software was used for the signal registration. EEG data were analyzed in EEGlab (Matlab Toolbox) ver. 13.2.2.b. Statistical analyses were performed in IBM SPSS Statistics v.23.

### EEG recording and data analysis

Continuous EEG data were registered with a frequency rate of 500 Hz. The signal was filtered with a 0.1–250 Hz bandpass filter. 64 electrodes were mounted on an elastic cap according to the 10–20 system. The FCz electrode served as an on-line reference. Impedance < 5 k Ohm was kept throughout the whole experiment. Prior to the statistical analyses, the EEG signal underwent an artifact subspace reconstruction (see: Mullen et al., 2013). An independent component analysis (ICA) was subsequently run to detect and reject artifactual components of non-brain origin. The signal was subsequently re-referenced to the averaged mastoid electrodes. Next, a bandpass filter was used (0.1–40 Hz, Butterworth infinite impulse response). The signal was then segmented separately for each of the big blocks (Space, Space-Time, Time, Random) into epochs from −200 to 1,000 ms with reference to the moment when the set of stimuli appeared on the screen. Only correct responses were considered for the Random segments and for predictable stimuli in the Space, Space-Time, and Time segments. After the baseline correction, automatic rejection was applied for signals exceeding ±60 μV. Next, the ERP components were defined according to the following steps.

For the P1 individual peak amplitude and its latency was found (separately for each subject) in the 80–140 ms time-window from electrodes PO7/O1 and PO8/O2. The amplitude was computed as average of ±5 sample points around the peak value. The signal was then averaged across participants to acquire the final P1 component.

The N2pc component was computed as the difference wave between the contra- and the ipsilateral signal, with reference to the target-containing visual hemifield, recorded from the PO7/PO8 electrodes. We chose the electrodes based on studies using similar paradigms (Mazza et al., 2007; Töllner et al., 2012a,b). Before averaging, we additionally applied the 8 Hz low-pass filter, according to the procedure proposed by Hackley et al. (2007). The difference waves were averaged separately for left and right targets, and the individual peak amplitudes as well as their latencies were defined in the 150–350 ms time-window after the presentation of the search display. Then, ±5 sample points were averaged to acquire the amplitude value, and the grand average was computed.

The sLRP component was computed similarly to the N2pc difference wave, but for the signal recorded from the C3/C4 electrodes. Then, the jackknife method was applied (Miller et al., 1998; Ulrich and Miller, 2001) i.e., the component for each subject was obtained by averaging the signal for all the participants except the person for which the component was computed. Next, for each person, peak amplitudes were found in the 200–600 ms time window after the visual stimuli presentation. Onset latency was defined as 50% of the peak onset value. The signal was then averaged across participants for the final sLRP component acquisition.

## Results

### Accuracy and reaction times

Reactions faster than 200 ms and slower than 1,000 ms were excluded from the analyses. Mean accuracy was 98.48% (SD = 0.58%). Only correct responses were included in the analyses. Out of all trials, 1.97% was excluded (2.02% in the Space block, 1.89% in the Space-Time block, 1.84% in the Time block, and 2.15% in the Random block).

### Verification of the implicit learning effects

For each big predictable block, a separate rmANOVA was run with a 10-level within-subject factor of “mini-block” for reaction time as the dependent variable.^1^ This aimed to verify (1) the tendency toward faster reactions from mini-blocks 1 to 6, (2) a RT increase in the “broken” mini-blocks from 7 to 9, and (3) an RT decrease between mini-blocks 9 (broken) and 10 (recovered). In case of violations of the sphericity assumption, the Greenhouse-Geiser correction was used for the values of the degrees of freedom in this and the following analyses.

**FIGURE 3:**
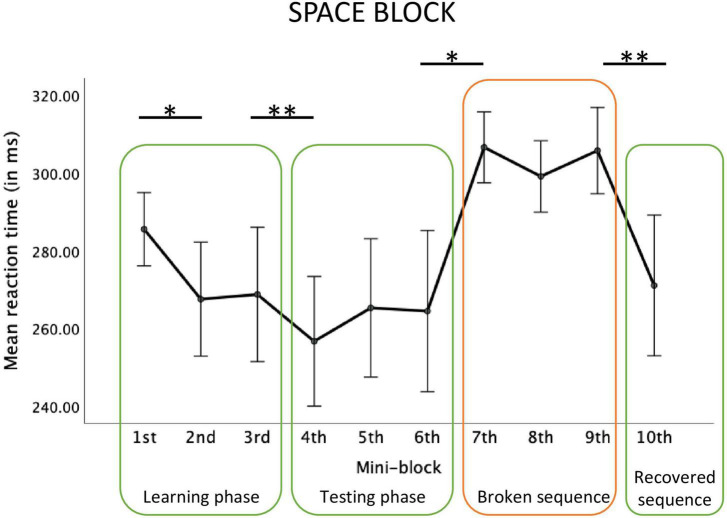
Reaction times in each mini-block of the big Space block. Error bars represent ±1 standard error. *Stands for *p* < 0.05, ^**^stands for *p* < 0.01.

For the Space block, the main effect of mini-block was significant, *F*(2.74, 38.42) = 6.00, *p* = 0.002; η_p_^2^ = 0.30, observed power = 0.92 see Figure 3). Repeated contrasts showed that there was a significant reaction time decrease in mini-block 2 relative to mini-block 1, *F*(1,14) = 5.09; *p* = 0.04, η_p_^2^ = 0.27, observed power = 0.56, a similar decrease between mini-blocks 3 and 4, *F*(1,14) = 11.16; *p* = 0.005, η_p_^2^ = 0.44, observed power = 0.87, a reaction time increase between mini-blocks 6 and 7, *F*(1,14) = 7.22; *p* = 0.02, η_p_^2^ = 0.34, observed power = 0.71, and a reaction time decrease between mini-blocks 9 and 10, *F*(1,14) = 9.19; *p* = 0.009, η_p_^2^ = 0.40, observed power = 0.80.

**FIGURE 4:**
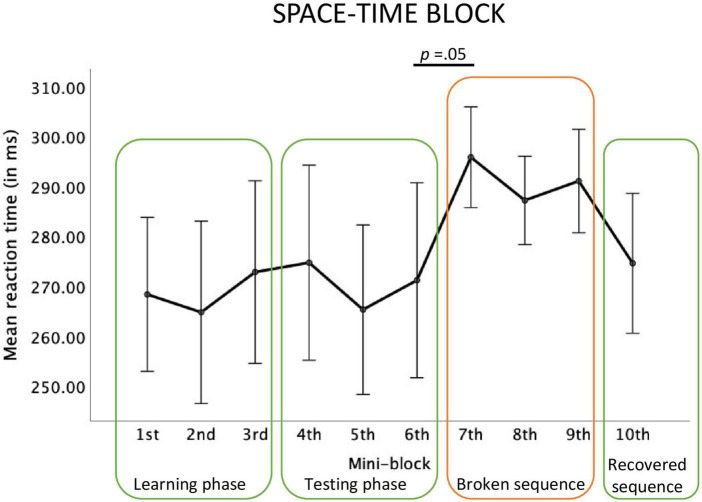
Reaction times in each mini-block of the big Time block.

For the Time block, the main effect of mini-block was significant, *F*(3.28, 45.94) = 5.94, *p* < 0.001; η_p_^2^ = 0.30, observed power = 0.95. However, the pattern of reaction times showed a gradual increase in reaction time instead of a learning curve (see Figure 4; the linear function was significant and best described the pattern of results, *F*(3.28, 45.94) = 13.49, *p* = 0.002; η_p_^2^ = 0.41).

**FIGURE 5:**
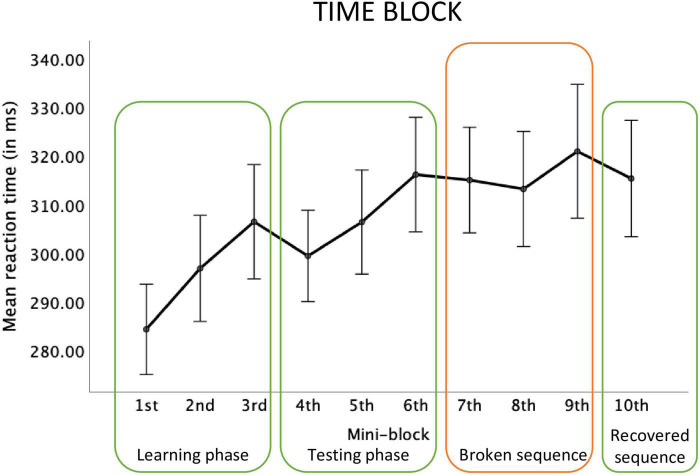
Reaction times in each mini-block of the big Space-Time block.

For the Space-Time block, the main effect of the mini-block was significant, *F*(2.68, 37.55) = 2.18, *p* = 0.03; η_p_^2^ = 0.13, observed power = 0.48. Repeated contrasts revealed a marginally significant effect of slower reactions in mini-block 7, compared to the mini-block 6, *F*(1,14) = 4.46; *p* = 0.05, η_p_^2^ = 0.24, observed power = 0.50. Although a relative reaction time increase between mini-blocks 9 and 10 is visible, this contrast did not reach significance, *F*(1,14) = 3.01; *p* = 0.10, η_p_^2^ = 0.18, observed power = 0.37, see Figure 5.

### Behavioral verification of the predictability effect

**FIGURE 6:**
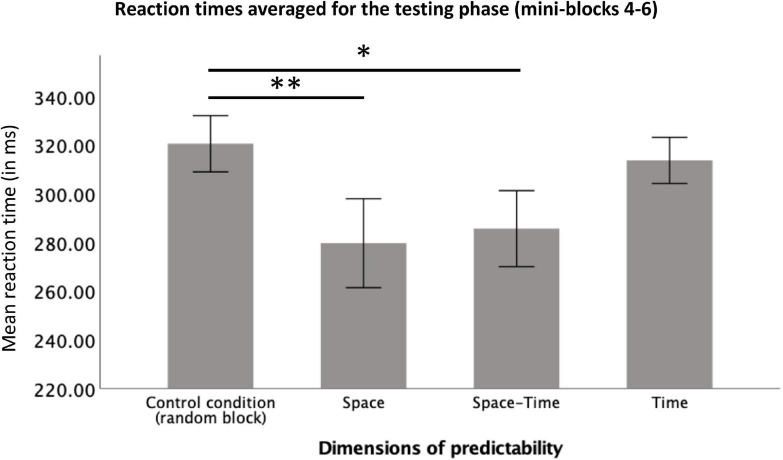
Reaction times averaged for the testing phase (mini-blocks 4–6) for each predictability condition. Error bars represent ±1 standard error. *Stands for *p* < 0.05, ^**^stands for *p* < 0.01.

**FIGURE 7:**
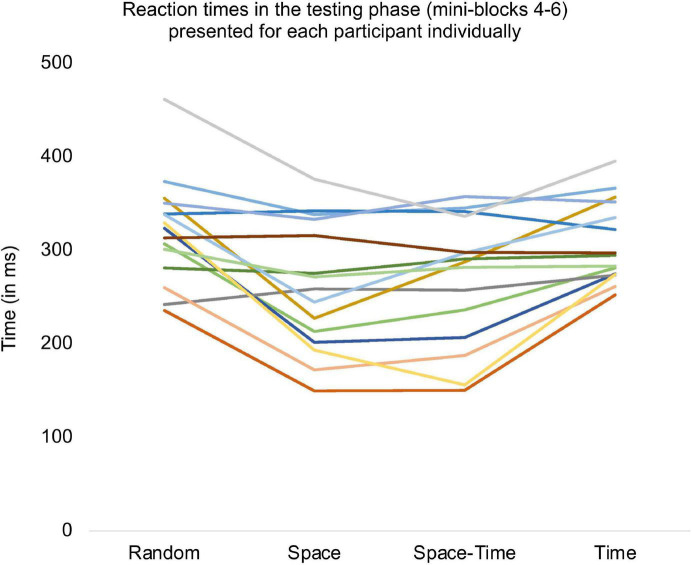
Reaction times averaged for all trials from the testing phase and presented separately for each subject.

To check whether participants reacted significantly faster after the initial learning phase in the big predictable blocks, relative to the same stage of procedural learning in the big random block, we averaged reaction times from mini-blocks 4–6 and compared them between the four big blocks. To this aim, we conducted a repeated-measures ANOVA with a 4-level “big block” factor (Random vs. Space vs. Space-Time vs. Time) for reaction time as the dependent variable. The number of trials (per condition) included in the analysis is provided in Table 1. The RT distributions did not differ significantly from the normal distribution (all *ps* > 0.11 in each condition). The main effect was significant, *F*(1.83,25.58) = 8.76, *p* = 0.002, η_p_^2^ = 0.38, observed power = 0.94, see Figure 6. Simple contrast analysis with the Random block as the reference category revealed shorter reaction times in the Space and Space-Time blocks *F*(1,14) = 10.53, *p* = 0.006, η_p_^2^ = 0.43, observed power = 0.85; *F*(1.14) = 8.99, *p* = 0.01, η_p_^2^ = 0.39; observed power = 0.80, respectively; simple contrast for the comparison with the Time block: *F*(1.14) = 0.49, *p* = 0.50, observed power = 0.10. Detailed descriptive statistics for the behavioral results are provided in Table 2. Reaction times of each individual subject in each condition are presented in Figure 7.

**TABLE 1:** Average number of trials and sequences plotted per participant in each big block, presented separately for the whole task and for the “testing” phase. Standard deviations are provided in parentheses.

Block	Trials	Sequences	Trials in the testing phase	Sequences in the testing phase
Random	282 (6)	70 (2)	141 (4)	35 (1)
Space	470 (11)	118 (3)	140 (6)	35 (1)
Space-Time	471 (10)	118 (2)	143 (2)	36 (0)
Time	471 (8)	118 (2)	142 (3)	35 (1)

One sequence equals four trials.

**TABLE 2:** Descriptive statistics for the behavioral results (reaction times in the testing phase, mini-blocks 4–6).

Block	*M* [ms]	*SD* [ms]
Random	312.88	42.76
Space	262.14	69.71
Space-Time	270.33	70.83
Time	307.30	39.73
Total	288.16	55.75

### Electrophysiological verification of the implicit learning facilitation mechanism

**FIGURE 8:**
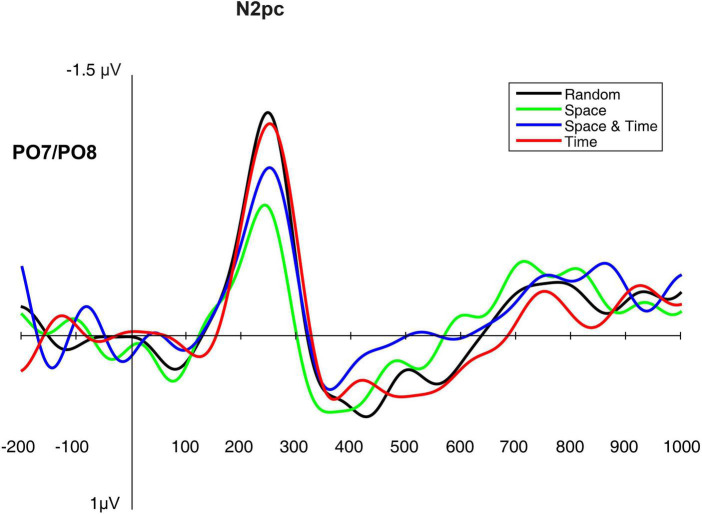
The N2pc component elicited in the testing phase (mini-blocks 4–6), presented separately for each big block.

Similarly to the above-described analyses, in order to compare the electrophysiological correlates of the processes hypothetically facilitated by the effects of implicit spatio-temporal learning, we analyzed several ERP components from the testing phase of each of the four big blocks (i.e., mini-blocks 4–6). Thus, separate rmANOVAs were conducted with a 4-level “big block” factor for the P1 and N2pc peak latencies and peak amplitudes, as well as for the sLRP onset latency and peak amplitude. Of all the expected effects, the main effect of block was significant for the N2pc peak latency, [*F*(3,42) = 4.82, *p* = 0.006, η_p_^2^ = 0.25, observed power = 0.87] as well as for the N2pc peak amplitude [*F*(3,42) = 4.23, *p* = 0.011, η_p_^2^ = 0.23, observed power = 0.82]. Simple contrast analysis with Random block as the reference category revealed shorter N2pc latency in the Space block, [*F*(1,14) = 10.56, *p* = 0.006, η_p_^2^ = 0.43, observed power = 0.85]; simple contrast effects for the comparisons with the Space-Time and Time blocks: *F*(1,14) = 0.17, *p* = 0.68, observed power = 0.07; *F*(1,14) = 0.51, *p* = 0.49, observed power = 0.10. An analogical simple contrast analysis for the N2pc peak amplitude showed a less negative deflection for spatially predictable targets [*F*(3,42) = 6.02, *p* = 0.028, η_p_^2^ = 0.30, observed power = 0.63]; simple contrast effects for the comparisons with the Space-Time and Time blocks: *F*(1,14) = 1.81, *p* = 0.29, observed power = 0.17; *F*(1,14) = 0.02, *p* = 0.88, observed power = 0.05. For the visualization of the N2pc component, see Figure 8. Detailed descriptive statistics are provided in Table 3. ANOVA results for the non-significant effects are presented in Table 4.

**TABLE 3:** Descriptive statistics for the significant electrophysiological results (N2pc peak latency and peak amplitude).

	Latency		Amplitude	
		
Block	*M* [ms]	*SD* [ms]	*M*[μV]	*SD* [μV]
Random	262.40	23.68	−1.48	1.08
Space	226.67	69.71	−0.92	0.88
Space-Time	249.33	43.75	−1.23	0.79
Time	261.60	36.82	−1.50	0.93
Total	250.00	43.49	−1.28	0.92

**TABLE 4:** Non-significant results of the repeated measure analysis of variance (rmANOVA) conducted for the event-related potential (ERP) components.

Component	Dependent variable	df_Num_	df_Den_	*F*	*p*	*F*_corrected_	*p*
P1	Peak latency	3	42	0.48	0.70	–	–
	Peak amplitude	3	42	1.67	0.19	–	–
sLRP	Latency (jackknife)	1.66	23.27	–	–	0.94	0.43
	Amplitude (jackknife)	1.89	26.52	–	–	2.52	0.07

## Discussion

The aim of the current study was to verify the effect of predictable spatial and temporal sequences on the efficiency of a localization task. We additionally wanted to unravel the neural mechanism underlying the expected behavioral facilitation. The study showed that implicitly learned predictability in spatial and spatiotemporal dimensions caused faster target localization in a visual search task. However, implicit sequential learning in the unidimensional temporal domain was not observed. In line with this finding, bidimensional predictability did not exhibit a greater facilitation effect over unidimensional spatial predictability. We did not find any perceptual or motor facilitation in the studied predictable spatio-temporal context.

In accordance with the tendency observed in previous research (Shin and Ivry, 2002; O’Reilly et al., 2008; Heideman et al., 2018), the facilitation effect of implicit learning was stronger for the spatial than for the temporal dimension. On the behavioral level, we observed the expected pattern of decreasing reaction times for spatially predictable targets during the learning and testing phases (mini-blocks 1–3 and 4–6) as well as a surprise effect (an increase in reaction times) when an unpredictable sequence appeared in mini-blocks 7–9. Reactions again became faster in the last mini block with the old predictable sequence. A typical implicit learning curve thus occurred (cf., e.g., Coomans et al., 2011; Guo et al., 2013; Meier and Cock, 2014). Moreover, as a result of comparing the reaction times from the testing phase in the Space block with analogous mini-blocks from the control condition, it turned out that this dimension of predictability significantly facilitated target localization. While this is not a surprising result, taking into account that the stimulus-reaction mapping correlated with the spatial dimension of predictability, the result concerning the electrophysiological underpinnings of this facilitation is not obvious. We found that faster motor reactions for spatially predictable targets were not because of faster motor response selection (no effect on the sLRP component). Instead, they were most probably due to a faster and less effortful attentional selection (shorter N2pc latency and less negative N2pc amplitude). Töllner et al. (2012a) also found a less negative N2pc amplitude but no effect on the N2pc latency for more vs. less frequent visual stimuli. According to the authors, this pattern indicates that equally rapid attentional selection of differently prevalent visual stimuli is possible due to signal enhancement for infrequent targets. In that sense, Töllner et al. (2012a) described the mechanism of compensating for uncertainty by cortical amplification (cf. Egner and Hirsch, 2005). Our study compared equally frequent stimuli with predictable vs. unpredictable locations. Therefore, one can ask whether the differences in the amplitude of the N2pc component are indicative of signal amplification for unpredictable stimuli or signal reduction for predictable stimuli. According to the assumptions of the predictive coding theory (Kok et al., 2012; Clark, 2013), and considering shorter N2pc latency, the second alternative seems more likely. Our result thus shows that the predictability of the response-relevant spatial dimension acts in the way of saving attentional resources. In other words, reduced N2pc amplitude and its shorter latency mean that less sensory evidence is needed to decide upon an accurate direction of attentional shift (cf. Töllner et al., 2012a). This is a new result in the context of the previously reported effects of spatial sequential learning; it may be related to the fact that most of the previous studies used procedures that did not involve visual search (Shin and Ivry, 2002; O’Reilly et al., 2008; Heideman et al., 2018, but see Coomans et al., 2011), or no electrophysiological components were recorded (Shin and Ivry, 2002; O’Reilly et al., 2008; Coomans et al., 2011; Heideman et al., 2018). Our finding is consistent with research on overt attention. McDonnell et al. (2014) showed that despite the lack of differences in the speed of directing the first saccade toward a predictable target, the detection of targets at predictable locations was faster compared to reactions to randomly exposed targets. The authors concluded that the perceptual and attentional–instead of oculomotor–processes might have been accelerated (see also Coomans et al., 2014). Therefore, the results of the present study complement the current state of knowledge on learning spatial sequences with the previously unreported effects of facilitated attentional selection in a visual-search paradigm (cf., Shin and Ivry, 2002; Abrahamse et al., 2010; Schwarb and Schumacher, 2012).

Regarding the lack of a temporal predictability effect, our findings comply with the previous reports by O’Reilly et al. (2008), who showed that a predictable sequence of time intervals alone does not facilitate target localization. The authors speculate that the intervals they used might not have been distinctive enough to sufficiently facilitate the perceptual task. Just as in the study by Los et al. (2017) who in fact observed successful temporal learning, the intervals in the current study had a similar difference value (i.e., 400 ms). Thus, the inappropriate difference value is not a likely explanation. An alternative reason for the lack of an effect in the study by O’Reilly et al. (2008) was the argument that the eight-element sequence was too long to be integrated into memory as a predictable chunk. In our research, we used a sequence of four elements, which is equal to the average working memory capacity (cf., Cowan, 2010). It can therefore be assumed that the sequence length in the current study was optimal. Thus, the following alternative explanations are plausible: (1) sequential learning does not occur for the temporal domain in the case of a perceptually demanding visual search task; (2) exhibition of the effects of implicit temporal SL requires even more perceptually challenging conditions, similarly to the case of spatial SL in a discrimination task (see: Coomans et al., 2011); (3) SL only occurs in spatially predictable conditions.

The first explanation can be considered with reference to the knowledge on the need for attentional resources for successful implicit learning. Conway (2020) states that the involvement of attention is not necessary for implicit learning of a sequence composed of elements that are in close proximity. However, it is required when a hidden pattern organizes elements that are not adjacent to each other (de Diego-Balaguer et al., 2016). Attentional resources must be also engaged for the effective implicit learning of global structures (Bekinschtein et al., 2009), as well as in the case of multi-modal sequences (e.g., visual and auditory or visual and motor) or in rule-based learning (Smith et al., 1998; Hendricks et al., 2013). It can be therefore concluded that our temporal sequences were not a subject of implicit learning, probably because the elements of the sequence were separated by temporally unpredictable events (i.e., the response time and the fixation dot). This design resulted from the EEG signal acquisition standards, which did not leave much room for modification. However, it is also possible that our sample size or the number of the sequence repetitions did not allow for a sufficient exposure of the temporal predictability effect (the power obtained for the comparison of RTs from the testing phase between Random and Time block was equal to 0.10). According to one of the statistical learning principles outlined by Conway (2020), more complex patterns lead to a lower level of learning (cf., Schiff and Katan, 2014), and thus they require more effort and/or time to be acquired. Nevertheless, future studies might take up the challenge of creating more optimal sequences that could at the same time be tested in the ERP paradigm. Spatial sequences, in turn, although composed of non-adjacent elements, entered attentional focus probably because of the nature of the localization task, thus resulting in successful learning.

With reference to the perceptual difficulty level hypothesis, Goschke (1998) demonstrated that the mechanism of sequential learning in the auditory domain varies depending on whether selective attention must be engaged or not. We thus expected similar effects in the visual domain. However, it could be that the localization task was not sufficiently demanding to expose behavioral benefits. Future studies should therefore test the effects of temporal sequences in perceptually more difficult, e.g., discrimination, tasks.

The third explanation is based on previous studies which demonstrated that even though predictable temporal sequences do not operate in isolation, they can enhance the effects of spatial (sequential) predictability (Shin and Ivry, 2002; O’Reilly et al., 2008). Such a conclusion cannot be drawn from our findings. Moreover, there was no acceleration of attentional selection in the Space-Time block, although this could be expected based on the effects of spatial predictability in the Space block. Therefore, did temporal sequences undermine the spatial predictability effects? It might be that the presence of temporal sequences was an attentionally more challenging situation in comparison to the random block, where the same time interval could be accidentally repeated in two or more consecutive trials. Faster reactions for such repetitive targets are known as the sequential effect, which never occurred in the case of the Time block.

### Conclusion and theoretical implications

By detecting shorter latency and lower amplitude of the N2pc component in a localization task we demonstrated that attentional selection in spatially predictable context is both easier and faster. This is in line with the predictive coding theory, according to which prediction attenuates neural activity related to the anticipated events (cf. Friston, 2005; Dale et al., 2012). Whether these effects replicate in other visual search tasks remains to be unraveled by future studies.

In the context of the unraveled impact of spatial sequential predictability on covert selective attention, the question about a similar facilitation effect of temporal sequential predictability remains to be answered. To fully elucidate this issue, future studies should test temporal sequences, where the elements of a sequence would not be separated by unpredictable time intervals. Additionally, looking at the results of the present study through the lens of the predictive coding theory, one can ask about the limitations of the human ability of (and the need for) monitoring complex temporal structures when covert selective attention is at play. Therefore, future research should take up the challenge of testing temporal and spatio-temporal sequential learning when the temporal dimension of predictability is response relevant. Furthermore, the use of virtual reality systems would allow creation of more ecologically valid conditions, where the utility of the spatio-temporal predictability could be truly revealed.

Even though our findings do not entirely support our primary hypotheses, they provide some critical theoretical implications, e.g., for the predictive coding theory (Clark, 2013). Swallow and Zacks (2008) demonstrated that predictions are primarily created in relation to behaviorally relevant feature dimensions, i.e., ones that are directly linked to the action required in a current situation. Given that the knowledge of temporal sequences was not successfully acquired, it might be that the process of hypothesis generation and verification does not occur on a global scale but is selective (in terms of domains that might be crucial for effective action preparation). This speculation is in opposition to the predictive coding framework, according to which perceptual and motor systems should not be considered separately but as a unified inferring mechanism (Adams et al., 2013). The function of these (perceptual and motor) mechanisms would be to predict sensory signals simultaneously in all domains: auditory, visual, somatosensorial, interoceptive, and proprioceptive (Adams et al., 2013). However, such a global scale inferring process may be restricted if the costs of prediction acquisition outweigh its potential benefits, as might be the case when monitoring a complex temporal sequence. More research is needed to unravel this controversy.

### Limitations

In general, studies on implicit learning demand a very cautious design, especially when electroencephalographic indexes are at play. Such research paradigm demands reconciliation of divergent goals, i.e., providing a sufficient learning period on one hand, and avoiding a tiresome testing time on the other hand. Given a relatively weak power for detecting the effect of temporal predictability in our experiment, future studies should consider testing a larger sample. With the aim to avoid excessive EEG signal contamination, a between-subjects design would allow inclusion of a longer learning period, without the costs of tiredness.

## Data availability statement

The raw data supporting the conclusions of this article will be made available by the authors, without undue reservation.

## Ethics statement

The studies involving human participants were reviewed and approved by the Ethics Committee of the Institute of Psychology at the John Paul II Catholic University of Lublin. The patients/participants provided their written informed consent to participate in this study.

## Author contributions

MSze conceived, designed, and performed the experiment, analyzed the data, and wrote the manuscript. PA programmed the procedures and analyzed the data. MSzu supervised the research and edited the manuscript. All authors reviewed the manuscript for submission.

## Footnotes

1 Hypothetically, RTs registered by the PsychoPy software might differ from the RTs extracted from the EEG data due to the fact that after the EEG artifacts’ rejection procedure a smaller number of trials could be included. Nevertheless, the analogous analyses conducted on the EEG-extracted data provided the same results. Thus, all subsequent behavioral analyses were conducted on the PsychoPy-extracted data.

## References

[B1] Abrahamse E. L. Jiménez L. Verwey W. B. Clegg B. A. (2010). Representing serial action and perception. *Psychon. Bull. Rev.* 17 603–623. 10.3758/PBR.17.5.603 21037157

[B2] Adams R. A. Shipp S. Friston K. J. (2013). Predictions not commands: Active inference in the motor system. *Brain Struct. Funct.* 218 611–643. 10.1007/s00429-012-0475-5 23129312 PMC3637647

[B3] Beck M. R. Hong S. L. Van Lamsweerde A. E. Ericson J. M. (2014). The effects of incidentally learned temporal and spatial predictability on response times and visual fixations during target detection and discrimination. *PLoS One* 9:e94539. 10.1371/journal.pone.0094539 24732965 PMC3986114

[B4] Bekinschtein T. A. Dehaene S. Rohaut B. Tadel F. Cohen L. Naccache L. (2009). Neural signature of the conscious processing of auditory regularities. *Proc. Natl. Acad. Sci. U.S.A.* 106 1672–1677. 10.1073/pnas.0809667106 19164526 PMC2635770

[B5] Chun M. M. Wolfe J. M. (1996). Just say no: How are visual searches terminated when there is no target present? *Cogn. Psychol.* 30 39–78. 10.1006/cogp.1996.0002 8635311

[B6] Clark A. (2013). Whatever next? Predictive brains, situated agents, and the future of cognitive science. *Behav. Brain Sci.* 36 181–204. 10.1017/S0140525X12000477 23663408

[B7] Coles M. G. H. (1989). Modern mind-brain reading: Psychophysiology, physiology, and cognition. *Psychophysiology* 26 251–269. 10.1111/j.1469-8986.1989.tb01916.x 2667018

[B8] Conway C. M. (2012). “Sequential learning,” in *Encyclopedia of the sciences of learning*, ed. Seel R. M. (New York, NY: Springer Publications), 3047–3050.

[B9] Conway C. M. (2020). How does the brain learn environmental structure? Ten core principles for understanding the neurocognitive mechanisms of statistical learning. *Neurosci. Biobehav. Rev.* 112 279–299. 10.1016/j.neubiorev.2020.01.032 32018038 PMC7211144

[B10] Conway C. M. Christiansen M. H. (2005). Modality-constrained statistical learning of tactile, visual, and auditory sequences. *J. Exp. Psychol. Learn. Mem. Cogn.* 31 24–39. 10.1037/0278-7393.31.1.24 15641902

[B11] Coomans D. Deroost N. Zeischka P. Soetens E. (2011). On the automaticity of pure perceptual sequence learning. *Conscious. Cogn.* 20 1460–1472. 10.1016/j.concog.2011.06.009 21741273

[B12] Coomans D. Vandenbossche J. Homblé K. Van Den Bussche E. Soetens E. Deroost N. (2014). Does consolidation of visuospatial sequence knowledge depend on eye movements? *PLoS One* 9:e103421. 10.1371/journal.pone.0103421 25089701 PMC4121143

[B13] Cowan N. (2010). The magical mystery four: How is working memory capacity limited, and why? *Curr. Dir. Psychol. Sci.* 19 51–57. 10.1177/0963721409359277 20445769 PMC2864034

[B14] Dale R. Duran N. Morehead R. (2012). Prediction during statistical learning, and im- plications for the implicit/explicit divide. *Adv. Cogn. Psychol.* 8 196–209. 10.5709/acp-0115-z 22723817 PMC3376885

[B15] Daltrozzo J. Conway C. M. (2014). Neurocognitive mechanisms of statistical-sequential learning: What do event-related potentials tell us? *Front. Hum. Neurosci.* 8:437. 10.3389/fnhum.2014.00437 24994975 PMC4061616

[B16] de Diego-Balaguer R. Martinez-Alvarez A. Pons F. (2016). Temporal attention as a scaffold for language development. *Front. Psychol.* 7:44. 10.3389/fpsyg.2016.00044 26869953 PMC4735410

[B17] Egner T. Hirsch J. (2005). Cognitive control mechanisms resolve conflict through cortical amplification of task-relevant information. *Nat. Neurosci.* 12 1784–1790. 10.1038/nn1594 16286928

[B18] Eimer M. (2013). “The time course of spatial attention: Insights from event-related brain potentials,” in *The Oxford handbook of attention*, eds Nobre A. C. Kastner S. (Oxford: Oxford University Press), 289–317. 10.1093/oxfordhb/9780199675111.013.006

[B19] Fiser J. Aslin R. N. (2001). Unsupervised statistical learning of higher-order spatial structures from visual scenes. *Psychol. Sci.* 12 499–504. 10.1111/1467-9280.00392 11760138

[B20] Friston K. (2005). A theory of cortical responses. *Philos. Trans. R. Soc. Lond. B Biol. Sci.* 360 815–836. 10.1098/rstb.2005.1622 15937014 PMC1569488

[B21] Fuster J. M. Bressler S. L. (2012). Cognit activation: A mechanism enabling temporal integration in working memory. *Trends Cogn. Sci.* 16 207–218. 10.1016/j.tics.2012.03.005 22440831 PMC3457701

[B22] Goschke T. (1998). “Implicit learning of perceptual and motor sequences: Evidence for independent systems,” in *Handbook of implicit learning*, eds Stadler M. A. Frensch P. (Thousand Oaks, CA: Sage), 401–444.

[B23] Guo X. Jiang S. Wang H. Zhu L. Tang J. Dienes Z. (2013). Unconsciously learning task-irrelevant perceptual sequences. *Conscious. Cogn.* 22 203–211. 10.1016/j.concog.2012.12.001 23318647

[B24] Hackley S. A. Miller J. (1995). Response complexity and precue interval effects on the lateralized readiness potential. *Psychophysiology* 32 230–241. 10.1111/j.1469-8986.1995.tb02952.x 7784531

[B25] Hackley S. A. Valle-Inclán F. (2003). Which stages of processing are speeded by a warning signal? *Biol. Psychol.* 64 27–45. 10.1016/S0301-0511(03)00101-7 14602354

[B26] Hackley S. A. Schankin A. Wohlschlaeger A. Wascher E. (2007). Localization of temporal preparation effects via trisected reaction time. *Psychophysiology* 44 334–338. 10.1111/j.1469-8986.2007.00500.x 17343715

[B27] Hasson U. Chen J. Honey C. J. (2015). Hierarchical process memory: Memory as an integral component of information processing. *Trends Cogn. Sci.* 19 304–313. 10.1016/j.tics.2015.04.006 25980649 PMC4457571

[B28] Heideman S. G. van Ede F. Nobre A. C. (2018). Temporal alignment of anticipatory motor cortical beta lateralisation in hidden visual-motor sequences. *Eur. J. Neurosci.* 48 2684–2695. 10.1111/ejn.13700 28921756 PMC6220967

[B29] Heinze H. J. Luck S. J. Mangun G. R. Hillyard S. A. (1990). Visual event-related potentials index focused attention within bilateral stimulus arrays. I. Evidence for early selection. *Electroencephalogr. Clin. Neurophysiol.* 75 511–527. 10.1016/0013-4694(90)90138-a 1693896

[B30] Hendricks M. Conway C. M. Kellogg R. T. (2013). Using dual-task methodology to dissociate automatic from nonautomatic processes involved in artificial grammar learning. *J. Exp. Psychol. Learn. Mem. Cogn.* 39 1491–1500. 10.1037/a0032974 23627281

[B31] Heuer H. Schmidtke V. Kleinsorge T. (2001). Implicit learning of sequences of tasks. *J. Exp. Psychol. Learn. Mem. Cogn.* 27:967. 10.1037/0278-7393.27.4.967 11486930

[B32] Howard J. H. Mutter S. A. Howard D. V. (1992). Serial pattern learning by event observation. *J. Exp. Psychol. Learn. Mem. Cogn.* 18 1029–1039. 10.1037/0278-7393.18.5.1029 1402708

[B33] Kleinsorge T. Schmidtke V. Gajewski P. Heuer H. (2003). The futility of explicit knowledge of a sequence of tasks. *Eur. J. Cogn. Psychol.* 15 455–469. 10.1080/09541440244000175

[B34] Kok P. Rahnev D. Jehee J. F. M. Lau H. C. De Lange F. P. (2012). Attention reverses the effect of prediction in silencing sensory signals. *Cereb. Cortex.* 22 2197–2206. 10.1093/cercor/bhr310 22047964

[B35] Los S. A. Kruijne W. Meeter M. (2017). Hazard versus history: Temporal preparation is driven by past experience. *J. Exp. Psychol. Hum. Percept. Perform.* 43 78–88. 10.1037/xhp0000279 27808547

[B36] Luck S. J. (2012). “Electrophysiological correlates of the focusing of attention within complex visual scenes: N2pc and related ERP components,” in *The Oxford handbook of event-related potential components*, eds Kappenman E. S. Luck S. J. (Oxford: Oxford University Press), 329–360.

[B37] Luck S. J. Hillyard S. A. (1994). Electrophysiological correlates of feature analysis during visual search. *Psychophysiology* 31 291–308. 10.1111/j.1469-8986.1994.tb02218.x 8008793

[B38] Luck S. J. Fuller R. L. Braun E. L. Robinson B. Summerfelt A. Gold J. M. (2006). The speed of visual attention in schizophrenia: Electrophysiological and behavioral evidence. *Schizophr. Res.* 85 174–195. 10.1093/oxfordhb/9780195374148.013.0161 16713184

[B39] Luck S. J. Heinze H. J. Mangun G. R. Hillyard S. A. (1990). Visual event-related potentials index focused attention within bilateral stimulus arrays. II. Functional dissociation of P1 and N1 components. *Electroencephalogr. Clin. Neurophysiol.* 75 528–542. 10.1016/0013-4694(90)90139-B 1693897

[B40] Mangun G. R. Hillyard S. A. (1991). Modulations of sensory-evoked brain potentials indicate changes in perceptual processing during visual-spatial priming. *J. Exp. Psychol. Hum. Percept. Perform.* 17 1057–1074. 10.1037/0096-1523.17.4.1057 1837297

[B41] Mayr U. (1996). Spatial attention and implicit learning of spatial and nonspatial sequences. *J. Exp. Psychol. Learn. Mem. Cogn.* 22 350–364. 10.1037//0278-7393.22.2.350 8901340

[B42] Mazza V. Turatto M. Umiltà C. Eimer M. (2007). Attentional selection and identification of visual objects are reflected by distinct electrophysiological responses. *Exp. Brain Res.* 181 531–536. 10.1007/s00221-007-1002-4 17602216 PMC2258005

[B43] McDonnell G. P. Mills M. McCuller L. Dodd M. D. (2014). How does implicit learning of search regularities alter the manner in which you search? *Psychol. Res.* 79 1–11. 10.1007/s00426-014-0546-8 24558017

[B44] Meier B. Cock J. (2014). Offline consolidation in implicit sequence learning. *Cortex* 57 156–166. 10.1016/j.cortex.2014.03.009 24861420

[B45] Miller J. Low K. (2001). Motor processes in simple, Go/No-Go, and choice reaction time tasks: A psychophysiological analysis. *J. Exp. Psychol. Hum. Percept. Perform.* 27 266–289. 10.1037/0096-1523.27.2.266 11318047

[B46] Miller J. Patterson T. Ulrich R. (1998). Jackknife-based method for measuring LRP onset latency differences. *Psychophysiology* 35 99–115. 10.1111/1469-8986.3510099 9499711

[B47] Miller J. Ulrich R. Rinkenauer G. (1999). Effects of stimulus intensity on the lateralized readiness potential. *J. Exp. Psychol. Hum. Percept. Perform.* 25 1454–1471. 10.1037/0096-1523.25.5.1454

[B48] Mullen T. Kothe C. Chi Y. M. Ojeda A. Kerth T. Makeig S. (2013). “Real-time modeling and 3D visualization of source dynamics and connectivity using wearable EEG,” in *Proceedings of the 35th annual international conference of the IEEE engineering in medicine and biology society* (Osaka: IEEE), 2184–2187. 10.1109/EMBC.2013.6609968 PMC4119601 24110155

[B49] Navon D. (1979). On the economy of the human-processing system. *Psychol. Rev.* 86 214–255. 10.1037/0033-295X.86.3.214

[B50] Nissen M. J. Bullemer P. (1987). Attentional requirements of learning: Evidence from performance measures. *Cogn. Psychol.* 19 1–32. 10.1016/0010-0285(87)90002-8

[B51] O’Reilly J. X. McCarthy K. J. Capizzi M. Nobre A. C. (2008). Acquisition of the temporal and ordinal structure of movement sequences in incidental learning. *J. Neurophysiol.* 99 2731–2735. 10.1152/jn.01141.2007 18322005

[B52] Praamstra P. Kourtis D. Kwok H. F. Oostenveld R. (2006). Neurophysiology of implicit timing in serial choice reaction-time performance. *J. Neurosci.* 26 5448–5455. 10.1523/JNEUROSCI.0440-06.2006 16707797 PMC6675318

[B53] Reber A. S. (1967). Implicit learning ofartificial grammars. *J. Verbal Learn. Verbal Behav.* 6 855–863. 10.1016/S0022-5371(67)80149-X

[B54] Saffran J. R. Aslin R. N. Newport E. L. (1996). Statistical learning by 8-month-old infants. *Science* 5294 1926–1928. 10.1126/science.274.5294.1926 8943209

[B55] Schiff R. Katan P. (2014). Does complexity matter? Meta-analysis of learner performance in artificial grammar tasks. *Front. Psychol.* 5:1084. 10.3389/fpsyg.2014.01084 25309495 PMC4174743

[B56] Schwarb H. Schumacher E. H. (2012). Generalized lessons about sequence learning from the study of the serial reaction time task. *Adv. Cogn. Psychol.* 8 165–178. 10.2478/v10053-008-0113-1 22723815 PMC3376886

[B57] Shin J. C. Ivry R. B. (2002). Concurrent learning of temporal and spatial sequences. *J. Exp. Psychol. Learn. Mem. Cogn.* 28 445–457. 10.1037/0278-7393.28.3.445 12018497

[B58] Smith E. E. Patalano A. L. Jonides J. (1998). Alternative strategies of categorization. *Cognition* 65 167–196. 10.1016/S0010-0277(97)00043-7 9557382

[B59] Smulders F. T. Y. Kok A. Kenemans J. L. Bashore T. R. (1995). The temporal selectivity of additive factor effects on the reaction process revealed in ERP component latencies. *Acta Psychol.* 90 97–109. 10.1016/0001-6918(95)00032-P 8525879

[B60] Sommer W. Leuthold H. Ulrich R. (1994). The lateralized readiness potential preceding brief isometric force pulses of different peak force and rate of force production. *Psychophysiology* 31 503–512. 10.1111/j.1469-8986.1994.tb01054.x 7972605

[B61] Swallow K. M. Zacks J. M. (2008). Sequences learned without awareness can orient attention during the perception of human activity. *Psychon. Bull. Rev.* 15 116–122. 10.3758/PBR.15.1.116 18605490

[B62] Töllner T. Rangelov D. Muller H. J. (2012a). How the speed of motor-response decisions, but not focal-attentional selection, differs as a function of task set and target prevalence. *Proc. Natl. Acad. Sci. U.S.A.* 109 E1990–E1999. 10.1073/pnas.1206382109 22733755 PMC3396490

[B63] Töllner T. Strobach T. Schubert T. Müller H. J. (2012b). The effect of task order predictability in audio-visual dual task performance: Just a central capacity limitation? *Front. Integr. Neurosci.* 6:75. 10.3389/fnint.2012.00075 22973208 PMC3438480

[B64] Ulrich R. Miller J. (2001). Using the jackknife-based scoring method for measuring LRP onset effects in factorial designs. *Psychophysiology* 38 816–827. 10.1111/1469-8986.3850816 11577905

[B65] Verwey W. B. Shea C. H. Wright D. L. (2014). A cognitive framework for explaining serial processing and sequence execution strategies. *Psychon. Bull. Rev.* 22 54–77. 10.3758/s13423-014-0773-4 25421407

[B66] Willingham D. B. Wells L. A. Farrell J. M. Stemwedel M. E. (2000). Implicit motor sequence learning is not represented purely in response locations. *Mem. Cogn.* 28 366–375. 10.1080/17470210902732130 10881554

[B67] Wolber M. Wascher E. (2005). The posterior contralateral negativity as a temporal indicator of visuo-spatial processing. *J. Psychophysiol.* 19 182–194. 10.1027/0269-8803.19.3.182

